# Effect of Instrumentation Level on Mental Health Subscale of Scoliosis Research Society Outcomes Questionnaire in Adolescent Idiopathic Scoliosis

**DOI:** 10.7759/cureus.14234

**Published:** 2021-04-01

**Authors:** Cem Albay, Mehmet Akif Kaygusuz

**Affiliations:** 1 Orthopaedics and Traumatology, Baltalimani Bone Diseases Research and Training Hospital, Istanbul, TUR

**Keywords:** adolescent idiopathic scoliosis, mental health, lowest instrumented vertebra, health-related quality of life, lumbar mobility, patient-reported outcome measures

## Abstract

Introduction

Adolescent idiopathic scoliosis (AIS) surgery aims to obtain a flexible and stable spine, correct axial rotation and halt curve progression by fusing the least number of motion segments. Longer fusions can improve deformity correction, but late decompensation and lumbar degeneration may occur. Even some daily issues can be problematic in some scoliosis patients. Reduction in mobility, segmental mobility adjacent to fusion, degeneration of junctional unfused levels and low-back pain (LBP) results in poor outcomes. It is reported that AIS patients have more mental and psychological problems including depression. Preoperative concerns due to deformity may continue postoperatively as instrumentation fuses motion segments. We wanted to present the relationship between the remaining mobile segments and Scoliosis Research Society-22r (SRS-22r) mental health (MH) scores of AIS patients.

Methods

It is a retrospective study of 110 posterior fusion AIS patients, age between 10 and 18 years, and followed-up for at least two years who filled SRS-22r forms both preoperatively and in postoperative second year (POY2). Patients are grouped according to the lowest instrumented vertebra (LIV) as LIV L5: group 1, LIV L4: group 2, LIV L3 and above: group 3.

Results

A statistically significant difference (SSD) was not found in preoperative and POY2 comparison of truncal shift (TTS), thoracic kyphosis (TK), lumbar lordosis (LL), sagittal balance (SB) in group 1; SB in group 2, and LL in group 3 (p>0.05). SSD was found in all other radiologic measurements of groups and in all patients. No SSD was found in function and MH in group 1, in function and pain in group 2 and in pain in group 3 and all patients (p>0.05). SSD was found in the remaining SRS-22r subscales. There was an SSD between groups 1 and 2 in terms of instrumentation level (p=0.013). SSD was found between groups 1 and 2 in preoperative Cobb angle (p=0.016). SSD was found between groups 2 and 3 in POY2 Cobb angle (p=0.025). SSD was found between groups 2 and 3 in POY2 apical vertebral translation (p=0.01). There was no SSD in other radiological parameters (p>0.05).

SSD was found between groups 1 and 2 (p=0.02) and between groups 1 and 3 (p=0.037) in terms of POY2 MH, but no SSD was found between groups 2 and 3 (p>0,05). There was no SSD in other preoperative or POY2 SRS-22r subscales (p>0.05).

Conclusion

More distal LIV is associated with a reduction of mobility and SRS scores. The self-image scores of groups were not statistically different. However, in group 1, MH was significantly lower. With the improvement of body images, patients start to worry about mobility instead of cosmesis. Higher depression has been reported in AIS patients. In POY2, there was no SSD between groups in terms of pain, function and satisfaction in addition to the self-image score as indicated in the literature.

We especially recommend that L5 LIV patients should receive psychiatric support in order to prevent anxiety, and inform and support them that they are not different in terms of pain, self-image and function scores compared to more proximal LIV patients, and also we recommend encouraging L5 LIV patients that POY2 SRS22-r pain, self-image, satisfaction, subtotal and total scores are improved. As reported in the literature which shows increased suicidal thoughts and depression in scoliosis patients; our findings regarding MH are important and considerable.

## Introduction

Adolescent idiopathic scoliosis (AIS) is a three-dimensional deformity with an angulation of ≥100 in the coronal plane, often accompanied by hyperkyphosis in the sagittal plane and rotation in the axial plane. Lenke type 5 is the main curvature of the lumbar/thoracolumbar region. According to Lenke's classification, structural curvatures should be fused, and non-structural curvatures should not [1]. The surgery aims to obtain a flexible and stable spine, correct axial rotation, maintain coronal and sagittal balance and halt curve progression by fusing the least number of motion segments [2]. Selective fusion can be applied to types 1, 2 and 5 curves. Even though improved deformity correction may be achieved with longer fusions, late sagittal decompensation and lumbar degeneration acceleration can be observed [3]. In the selective fusion of a structural thoracic curve, the lumbar curve is not fused to preserve postoperative mobility and flexibility despite the possibility of providing more correction. On the other hand, the fusion of the structural lumbar curve in lumbar scoliosis will decrease postoperative flexibility and mobility [4].

The lumbar spine is more mobile than the thoracic spine due to different orientations of facet joints and not being fixed with ribs [4]. Ordinary issues that a person with a healthy spine can easily apply in daily life such as tying shoelaces, leaning forward, picking up a falling object from the ground can be problematic in some scoliosis patients. Scoliosis surgery is fusion surgery. Accordingly, the movement previously shared by all spines is shared by a small number of vertebrae that remain only distal to the fusion site after surgery. This may lead to movement restrictions and premature degeneration of remaining discs due to exposure to higher loads [3].

Reduction in overall trunk mobility and physical functioning, segmental mobility adjacent to fusion, degeneration of junctional unfused levels of the spine and low-back pain (LBP) results in poor outcomes [5,6]. Before scoliosis surgery, type 5 patients are informed about possible postoperative movement limitations and possible adjacent segment disease that may develop over the years, possible discopathies, pelvic asymmetry or implant failure, such that the instrumentation may need to be extended distally. During adolescence, adolescents focus on their bodies. The adolescents undergo psychological and physical changes; it is clear that the AIS patient will also have anxiety related to the body. It has been reported in the literature that scoliosis patients have more mental and psychological problems including depression compared to their healthy peers [7]. Concerns due to the deformity in the preoperative period may also continue postoperatively in patients with lumbar scoliosis, as instrumentation fuses the moving vertebrae. In this study, we wanted to present the relationship between the number of remaining mobile segments after surgery and SRS-22r mental health (MH) scores, which reflect the postoperative anxiety status and psychology of AIS patients.

## Materials and methods

The study was carried out retrospectively with 110 patients in a tertiary reference orthopedics and traumatology training and research hospital after Institutional Review Board approval (8.6.18/30). The study was carried out according to the Declaration of Helsinki. Written informed consent was obtained from patients or their next caretakers. Inclusion criteria and exclusion criteria are presented in Table 1.

**Table 1:** Inclusion and exclusion criteria of the patients AIS: adolescent idiopathic scoliosis, SRS: Scoliosis Research Society, POY2: postoperative second year.

Inclusion criteria	Exclusion criteria
Lenke type 5 AIS patients	Non-idiopathic scoliosis (i.e., congenital, neuromuscular, etc.)
Only posterior fusion with pedicle screw-only constructs	Infantile, juvenile or adult idiopathic scoliosis
Operated between 2011 January and 2019 January	Intraoperative or postoperative complications (i.e., neurologic deficit, infection, pseudoarthrosis, implant-related insufficiency)
Ages between 10 and 18 years	More than one operation
Followed-up for at least two years	Other than only posterior fusion surgery
No previous spinal surgery	Absence of preoperative and/or POY2 SRS-22r form(s)
Presence of SRS-22r forms both preoperatively and in the POY2	Spinal trauma after surgery
	Absence of preoperative radiographs
	Patients who were out-of follow-up
	History of previous spinal surgery before fusion

In the surgical evaluation of the patients, the progression of the curve, presence of pain, function, residual growth pattern should be evaluated. Preoperative and postoperative conditions of AIS patients are evaluated with SRS-22r forms. An increase in SRS-22r scores indicates that the patient's condition improves, and a decrease indicates that it worsens [2]. SRS-22r evaluates patients under five subscales: function, pain, self-image, MH, satisfaction/dissatisfaction with management. Among these, MH evaluates the psychiatric status and anxiety of the patients. Questions number 3, 7, 13, 16 and 20 evaluates the MH subscale. Being a nervous person, feeling so down in the dumps that nothing could cheer the patient up, feeling calm and peaceful, feeling downhearted and blue, and being a happy person during the past six months are assessed and scored from 1 to 5 by SRS-22r. Radiographs and patient files were evaluated retrospectively and operative age, gender and Risser stage were determined.

Patients are grouped according to the lowest instrumented vertebra (LIV). If LIV is L5, the only mobile segment is L5-S1. If LIV is L4, there are two mobile segments (L4-5 and L5-S1). If LIV is L3, there are three mobile segments (L3-4, L4-5 and L5-S1). One mobile segment (LIV: L5) was grouped as group 1, two mobile segments (LIV: L4) were grouped as group 2, three and more mobile segments (LIV: L3 or above) were grouped as group 3. End vertebras, instrumentation levels, operation durations, preoperative and postoperative second year (POY2) Cobb angles, apical vertebral translation (AVT; distance between central sacral vertical line (CSVL) and the center of apical vertebrae of TL/L curve), coronal balance (CB), thoracic truncal shift (TTS; distance between C7 plumb line and CSVL), T5-T12 thoracic kyphosis angles (TK), L1-S1 lumbar lordosis angles (LL), sagittal balance (SB), and LIV tilt angles (LIVTA) were measured in plain scoliosis anterior-posterior and lateral radiographs and are presented Table 1. Preoperative and POY2 SRS-22r subscales, subtotal score and total score are presented in Table 2. Radiologic parameters were measured twice by two authors in four weeks intervals and the mean of the values was evaluated.

**Table 2:** Radiographic measurements of groups and all patients, given as mean (±SD)/median (min-max) Preop: preoperative, POY2: postoperative second year, CB: coronal balance, AVT: apical vertebral translation, TTS: truncal thoracic shift, TK: thoracic kyphosis, LL: lumbar lordosis, SB: sagittal balance, LIVTA: lowest instrumented vertebra tilt angle.

	Group 1 (n= 10)		Group 2 (n= 45)		Group 3 (n= 55)		All patients (n= 110)	
	Preop	POY2	Preop	POY2	Preop	POY2	Preop	POY2
Cobb	57.1 (±7.3)	11.7 (±6.5)	47.4 (±10)	10 (±5.8)	50.9 (±9.9)	13.7 (±7.8)	51.2 (27.3–83.6)	10.4 (1.3–39.3)
	p<0.0001		p<0.0001		p<0.0001		p<0.0001	
CB	21.5 (±13.9)	10.7 (±6.2)	17.96 (2.6-48)	6.25 (0.1–18.3)	21.1 (±13.3)	7.56 (0–34)	18.2 (2.3–60.7)	7.5 (0–34)
	p=0.009		p<0.0001		p<0.0001		p<0.0001	
AVT	48.5 (±22.1)	10 (±2.6)	35.67 (9.7–83.4)	6.9 (0–25.2)	42.2 (23.3–65.2)	12.4 (±7.1)	40.2 (9.7–87.3)	8.9 (0–29.3)
	p<0.0001		p<0.0001		p<0.0001		p<0.0001	
TTS	20.6 (±16.8)	10.6 (±4.2)	10.9 (0–42.9)	6.5 (0.5–29.6)	13.2 (2.7–48.1)	7.9 (±6)	12.6 (0–48.1)	7.6 (0–29.6)
	p=0.089		p=0.001		p<0.0001		p<0.0001	
TK	19.7 (±8.9)	26.5 (±8.2)	27.1 (±10.8)	32.4 (±9.5)	24.7 (±11.8)	28.6 (±12.6)	25.4 (±11.3)	29.9 (±11.2)
	p=0.095		p=0.005		p=0.013		p<0.0001	
LL	−55.2 (±13.4)	−51.5 (±13.3)	−57.7 (±11.9)	−48.7 (±12.2)	−54.9 [(−74.4)–(97.5)]	−42.6 (±15.9)	-57.1 [(−80.5)–(97.5)]	46.8 [(−77)–(35.4)]
	p=0.41		p<0.0001		p=0.051		p<0.0001	
SB	9.2 (±25.1)	12.4 (±19.9)	−19.5 [(−60.2)–(40.6)]	−2.6 (±21.3)	−7.7 (±38.7)	2.7 (±19.8)	−8.6 (±34.3)	1.4 (±20.7)
	p=0.753		p=0.055		p=0.041		p=0.005	
LIVTA	17.4 (±20.3)	1.3 (±7.2)	22.5 [(−24.3)–(40.1)]	4.9 (±6.4)	23.5 [(−37.1)–(42.7)]	3.4 (±7.2)	23.1 [(−37.1)–(43.5)]	3.9 (±6.9)
	p=0.012		p<0.0001		p<0.0001		p<0.0001	

Statistical analysis was performed using SPSS 22 software (Chicago, IL). Quantitative variables were given as mean (±SD) and median (IQR), whereas qualitative variables were presented as frequencies. Kolmogorov-Smirnov and Shapiro Wilk tests were performed for evaluation of normality. One-way ANOVA was used for comparison of normally distributed variables. Tukey’s multiple comparison test was used for comparisons in subgroups. Within-group differences between preoperative and postoperative values were evaluated using paired t-test. Alpha level was set at less than 0.05 to declare significance and p-value <0.001 was determined to be very significant.

## Results

Our study was carried out with 110 patients. One hundred one patients were female and nine patients were male. There was no statistical difference between groups in terms of gender (p>0.005). No significant difference was found in terms of preoperative and postoperative comparison of TTS, TK, LL, SB in group 1; SB in group 2, and LL in group 3 (p>0.05). A statistically significant difference was found in all remaining radiographic measurements of groups and in all patients' measurements in preoperative and postoperative comparison (Table 2). In terms of preoperative and postoperative comparison of SRS-22r subscales, no statistically significant difference was found in terms of function and MH in group 1, in terms of function and pain in group 2 and in terms of pain in group 3 and all 110 patients (p>0.05). A statistically significant difference was found in the remaining SRS-22r subscales comparisons (Table 3).

**Table 3:** SRS-22r scores of groups and all patients, given as mean (±SD)/median (min-max) Preop: preoperative, POY2: postoperative second year, F: function, P: pain, SI: self-image, MH: mental Health, SwM: satisfaction with management, Sbt: subtotal, T: total.

	Group 1 n= 10		Group 2 n= 45		Group 3 n= 55		All patients n= 110	
	Preop	POY2	Preop	POY2	Preop	POY2	Preop	POY2
F	15.6 (±2.2)	16.4 (±1.8)	16 (11−18)	16 (13−19)	15 (9−19)	16 (13−19)	15.5(9−19)	16(13−19)
	p=0.399		p=0.067		p=0.04		p=0.004	
P	17.6 (±4.3)	19.9 (±2.5)	19 (12−25)	19 (15−22)	19 (13−25)	20 (11−22)	19 (12−25)	19(11−23)
	p=0.034		p=0.843		p=0.444		p=0.364	
SI	12.1 (±2.9)	18.9 (±2.8)	13 (7−23)	19.8 (±2.8)	13 (6−23)	20 (11−25)	13 (6−23)	20 (11−25)
	p=0.001		p<0.0001		p<0.0001		p<0.0001	
MH	15 (14−16)	16 (13−16)	15 (14−17)	16 (15−17)	16 (14−17)	16 (14−17)	15 (14−17)	16(13−17)
	p=0.591		p<0.0001		p=0.004		p<0.0001	
SwM	6.6 (±1.5)	8.5 (7−10)	6 (5−10)	9 (5−10)	7 (2−10)	9 (4−10)	6.5 (2−10)	9 (4−10)
	p=0.002		p<0.0001		p<0.0001		p<0.0001	
Sbt	60.4 (±7.7)	70.5 (±4.4)	63.7 (±7.6)	70.6 (±4.9)	65 (45−79)	72 (59−79)	65 (45−79)	72 (59−79)
	p=0.007		p<0.0001		p<0.0001		p<0.0001	
T	67 (±7.2)	79.3 (±5.2)	70.5 (±7.6)	79.4 (±6)	70.3 (±8)	81 (64−93)	70.1 (±7.8)	80.5 (64−93)
	p=0.002		p<0.0001		p<0.0001		p<0.0001	

In the analysis between groups, there was a statistically significant difference between groups 1 and 2 in terms of instrumentation level (p=0.013). A statistically significant difference was found between groups 1 and 2 in terms of preoperative Cobb angle (p=0.016). A statistically significant difference was found between groups 2 and 3 in terms of postoperative Cobb angle (p=0.025). A statistically significant difference was found between groups 2 and 3 in terms of postoperative AVT (p=0.01). There was no statistically significant difference in terms of other radiological parameters (p>0.05). A statistically significant difference was found between groups 1 and 2 (p=0.02) and between groups 1 and 3 (p=0.037) in terms of postoperative MH, but no significant difference was found between groups 2 and 3 (p>0.05). There was no statistically significant difference in terms of other preoperative or postoperative SRS-22r domains (p>0.05).

## Discussion

Although many causes such as neuromuscular diseases, hereditary syndromes, congenital anomalies of the spine, leg length discrepancies, disc hernias, spinal tumors, chest wall anomalies are among etiological causes of scoliosis, the cause for scoliosis cannot be determined in the majority of patients. The aim of AIS surgery is to obtain a flexible and stable spine and halt curve progression. While curves are classified and treated according to Lenke classification, structural curvatures are included in the fusion area, but non-structural curvatures are not included. LIV's distal disc angel should be in less than a 10° tilt to obtain a straight spine [8]. Wang et al. reported that a preoperative LIVTA of more than 25° is associated with postoperative coronal decompensation [9].

Rose and Lenke reported that the lumbar level at the most proximal point intersected by CSVL on neutral AP radiographs should be the LIV [10]. Hamzaoglu et al. redefined LIV as the most proximal vertebra intersected by CSVL on concave bending radiographs but not on neutral AP radiographs [11]. Davis et al. defined traction under general anesthesia X-rays for rigid curves above 65° to determine the LIV [12]. Wang et al. reported that the greatest correction occurs when LIV is below lumber apical vertebra, even though stopping at lumber apical vertebra may preserve more lumbar mobility and growth potential [9]. Aaro and Ohlen showed a gradual loss of flexion when LIV moves inferior from T12 to L4 and the stress in the remaining mobile segments increases. They recommended preserving the L3−L4 motion segment [13]. Although longer spinal fusions have been shown to result in greater deformity correction, patients experienced an increased risk of lumbar degeneration potential and reduced flexibility.

Deformity-flexibility quotient (DFQ) is defined to identify the primary two goals of AIS surgery and is calculated by dividing the residual coronal lumbar deformity by the number of distal motion segments that are not fused. “DFQ<4” will occur as a result of the presence of more mobile lumbar segments with less postoperative deformities will be consistent with higher HRQoL results in patients [2].

Previous studies mainly reported proximal complications of Lenke 5C patients [14]. By the way, the distal progression of deformity occurs in 2% to 51% of Lenke 5C patients [15]. It may result in coronal imbalance and the patient may need revision surgery. Increased back pain and anxiety regarding recurved spine are reported. More distal LIV is associated with the reduction of spinal mobility [16]. Ohashi et al. reported that a significant reduction in flexion is related to poor SRS-22r pain, function, satisfaction, and total scores at a 10-year follow-up. Better results have been reported at 10 years postoperatively with surgeries leaving more mobile segments [16]. With more caudal LIV, rate of reduction in spinal mobility and HRQoL. The rate of return to athletic activity after posterior spinal fusion statistically decreases when LIV moves inferior from T11 to L4 [17]. Danielsson and Nachemson showed significant correlations with better HRQoL scores and more lumbar mobility 20 years postoperatively [18].

Previous literature has focused on curve correction rates and preservation of lumbar mobile segments, although studies examining the effect of LIV on MH are insufficient in literature [19]. Existing studies took L2 or L3 and above as one group. Studies generally evaluated patients with thoracic scoliosis or considered both thoracic and lumbar scoliosis together. In studies examining lumbar scoliosis, L4 and L5 were evaluated as lower lumbar levels and evaluated in one group. Ginsburg et al. evaluated L4 or L5 as lower LIV group and L3 and above as higher LIV group. LBP of groups were 53% and 38%, respectively [20]. Shu et al. selected L3 as the LIV in all patients [15]. In the study of Ohashi et al., patients with LIV with L2 and its distal were evaluated as one group, and patients with L1 and proximally as the other group; there was no difference in postoperative MH scores between the groups [16]. In other words, patients with LIV L5 and L2 patients were evaluated in the same group. However, LIV L5 patients have only one mobile segment. These patients consider their current surgery as their last chance and live with the anxiety of being at risk of fusion of the remaining single mobile segment in case of a possible complication. In our study, we examined the effect of the mobile segment on HRQoL by evaluating patients with L3 and above, L4 and L5 separately. In this manner, we considered the patients’ concerns as Marks et al. reported that postoperative hypermobility at distal unfused segments causes LBP in adulthood and trunk rigidity [21].

At the end of our study, we saw that the self-image scores of patient groups with different mobile segments were not statistically different. However, in the patient group with LIV L5, MH scores were significantly lower than in other groups. Although the most common complaint of patients who underwent surgery for Scoliosis is cosmetic reasons [4], self-image scores of the Lenke type 5 patients improve postoperatively in all groups; therefore, with the improvement of body images of patients, they start to worry about their mobility instead of cosmesis. AIS patients have been shown to be unhappier with their lives with higher depression [22]. Payne et al. showed that self-reported “scoliosis" is an independent risk factor for suicidal thoughts and worries and concerns regarding body development and peer interactions [23]. Roberts et al. found worse scores in the MH subscale in females and the majority of our study group consists of females [24].

Evaluation of lumbar mobility and HRQoL suggests that the outcome of L3 fusions is similar to that of shorter fusion, but below L4 fusion, there is less lumbar mobility and a poorer function score [25]. Sanchez-Raya et al. also grouped patients LIV T12-L1-L2, LIV L3 and LIV L4-L5-S1 and found outcomes of LIV L3 similar to the more proximal LIV group [25]. Functional loss and a higher incidence of disc degeneration and LBP are reported in the literature [2]. Bartie et al. found higher LBP intensity in a minimum 10-year follow-up in LIV L4 patients [26]. Even though Danielsson et al. and Helenius et al. showed no difference in terms of function as our study, the aim of surgery is to leave as many mobile segments as possible while maintaining CB [18,27].

In our study in POY2, there was no difference between groups in terms of pain, function and satisfaction subscales in addition to self-image score as indicated in literature [4]. In other words, these patients agreed that the treatment that should be applied to them was applied. There was no difference between groups in preoperative MH scores, but only in the postoperative MH scores, showing that they had psychological concerns about future outcomes depending on LIV level. As stated by Newton “A balanced and mobile lumbar spine without curve progression is better than a straight and stiff lumbar spine” [2]; LIV should be placed as proximal as possible to preserve more mobile segments and as distal as possible to avoid imbalance [28].

As limitations, preoperative lumbar flexibility of patients was not evaluated. Flexibility in male and female gender can be different. This is a single-center study that is composed of a single race with similar cultural features. Cultural differences, age and previous bracing may affect SRS-22r scores [29]. The socioeconomic background could not be considered in this study because of the retrospective design. Multicentric prospective studies with a longer follow-up period may be presented in the future.

## Conclusions

We especially recommend that L5 LIV patients should receive psychiatric support in order to prevent postoperative anxiety, and inform and support them that they are not different in terms of postoperative pain, self-image and function scores compared to patients with LIV at more proximal levels, and also, we recommend encouraging L5 LIV patients that postoperative SRS22-r pain, self-image, satisfaction, subtotal and total scores are improved by surgery. As reported in the literature which shows increased suicidal thoughts and depression in scoliosis patients; our findings regarding mental health are important and considerable.

## References

[1] Lenke LG, Edwards CC II, Bridwell KH (2003). The Lenke classification of adolescent idiopathic scoliosis: how it organizes curve patterns as a template to perform selective fusions of the spine. Spine (Phila Pa 1976).

[2] Newton PO, Upasani VV, Bastrom TP, Marks MC (2009). The deformity-flexibility quotient predicts both patient satisfaction and surgeon preference in the treatment of Lenke 1B or 1C curves for adolescent idiopathic scoliosis. Spine (Phila Pa 1976).

[3] Tambe AD, Panikkar SJ, Millner PA, Tsirikos AI (2018). Current concepts in the surgical management of adolescent idiopathic scoliosis. Bone Joint J.

[4] Uehara M, Takahashi J, Ikegami S (2019). Correlation of lower instrumented vertebra with spinal mobility and health-related quality of life after posterior spinal fusion for adolescent idiopathic scoliosis. Clin Spine Surg.

[5] Fan H, Wang Q, Huang Z, Sui W, Yang J, Deng Y, Yang J (2016). Comparison of functional outcome and quality of life in patients with idiopathic scoliosis treated by spinal fusion. Medicine (Baltimore).

[6] Marks MC, Bastrom TP, Petcharaporn M (2015). The effect of time and fusion length on motion of the unfused lumbar segments in adolescent idiopathic scoliosis. Spine Deform.

[7] Han J, Xu Q, Yang Y, Yao Z, Zhang C (2015). Evaluation of quality of life and risk factors affecting quality of life in adolescent idiopathic scoliosis. Intractable Rare Dis Res.

[8] Ando K, Imagama S, Ito Z (2016). Predictive factors for a distal adjacent disorder with L3 as the lowest instrumented vertebra in Lenke 5C patients. Eur J Orthop Surg Traumatol.

[9] Wang Y, Bünger CE, Zhang Y, Wu C, Li H, Dahl B, Hansen ES (2013). Lowest instrumented vertebra selection for Lenke 5C scoliosis: a minimum 2-year radiographical follow-up. Spine (Phila Pa 1976).

[10] Rose PS, Lenke LG (2007). Classification of operative adolescent idiopathic scoliosis: treatment guidelines. Orthop Clin North Am.

[11] Hamzaoglu A, Talu U, Tezer M, Mirzanli C, Domanic U, Goksan SB (2005). Assessment of curve flexibility in adolescent idiopathic scoliosis. Spine (Phila Pa 1976).

[12] Davis BJ, Gadgil A, Trivedi J, Ahmed el-NB (2004). Traction radiography performed under general anesthetic: a new technique for assessing idiopathic scoliosis curves. Spine (Phila Pa 1976).

[13] Aaro S, Ohlén G (1983). The effect of Harrington instrumentation on the sagittal configuration and mobility of the spine in scoliosis. Spine (Phila Pa 1976).

[14] Kwan MK, Chiu CK, Chan TS, Abd Gani SM, Tan SH, Chan CY (2018). Flexibility assessment of the unfused thoracic segments above the "potential upper instrumented vertebrae" using the supine side bending radiographs in Lenke 5 and 6 curves for adolescent idiopathic scoliosis patients. Spine J.

[15] Shu S, Bao H, Zhang Y (2020). Selection of distal fusion level for Lenke 5 curve: does the rotation of the presumed lower instrumented vertebra matter?. Spine (Phila Pa 1976).

[16] Ohashi M, Bastrom TP, Marks MC, Bartley CE, Newton PO (2020). The benefits of sparing lumbar motion segments in spinal fusion for adolescent idiopathic scoliosis are evident at 10 years postoperatively. Spine (Phila Pa 1976).

[17] Fabricant PD, Admoni S, Green DW, Ipp LS, Widmann RF (2012). Return to athletic activity after posterior spinal fusion for adolescent idiopathic scoliosis: analysis of independent predictors. J Pediatr Orthop.

[18] Danielsson AJ, Nachemson AL (2003). Back pain and function 23 years after fusion for adolescent idiopathic scoliosis: a case-control study-part II. Spine (Phila Pa 1976).

[19] Chang DG, Yang JH, Suk SI (2017). Importance of distal fusion level in major thoracolumbar and lumbar adolescent idiopathic scoliosis treated by rod derotation and direct vertebral rotation following pedicle screw instrumentation. Spine (Phila Pa 1976).

[20] Ginsburg HH, Goldstein LA, Robinson SC, Haake PW, Devanny JR, Chan DP, Suk SI (1979). Back pain in postoperative idiopathic scoliosis: long-term follow-up study. Spine.

[21] Marks M, Newton PO, Petcharaporn M (2012). Postoperative segmental motion of the unfused spine distal to the fusion in 100 patients with adolescent idiopathic scoliosis. Spine (Phila Pa 1976).

[22] Freidel K, Petermann F, Reichel D, Steiner A, Warschburger P, Weiss HR (2002). Quality of life in women with idiopathic scoliosis. Spine (Phila Pa 1976).

[23] Payne WK III, Ogilvie JW, Resnick MD, Kane RL, Transfeldt EE, Blum RW (1997). Does scoliosis have a psychological impact and does gender make a difference?. Spine (Phila Pa 1976).

[24] Roberts DW, Savage JW, Schwartz DG (2011). Male-female differences in Scoliosis Research Society-30 scores in adolescent idiopathic scoliosis. Spine (Phila Pa 1976).

[25] Sanchez-Raya J, Bago J, Pellise F, Cuxart A, Villanueva C (2012). Does the lower instrumented vertebra have an effect on lumbar mobility, subjective perception of trunk flexibility, and quality of life in patients with idiopathic scoliosis treated by spinal fusion?. J Spinal Disord Tech.

[26] Bartie BJ, Lonstein JE, Winter RB (2009). Long-term follow-up of adolescent idiopathic scoliosis patients who had Harrington instrumentation and fusion to the lower lumbar vertebrae: is low back pain a problem?. Spine (Phila Pa 1976).

[27] Helenius I, Remes V, Yrjönen T, Ylikoski M, Schlenzka D, Helenius M, Poussa M (2002). Comparison of long-term functional and radiologic outcomes after Harrington instrumentation and spondylodesis in adolescent idiopathic scoliosis: a review of 78 patients. Spine (Phila Pa 1976).

[28] Erdem MN, Karaca S, Korkmaz MF, Enercan M, Tezer M, Kara AN, Hamzaoglu A (2018). Criteria for ending the distal fusion at the L3 vertebra vs. L4 in surgical treatment of adolescent idiopathic scoliosis patients with Lenke type 3c, 5c, and 6c curves: results after ten years of follow-up. Cureus.

[29] Watanabe K, Lenke LG, Bridwell KH (2007). Cross-cultural comparison of the Scoliosis Research Society Outcomes Instrument between American and Japanese idiopathic scoliosis patients: are there differences?. Spine (Phila Pa 1976).

